# Phenotyping of Left and Right Ventricular Function in Mouse Models of Compensated Hypertrophy and Heart Failure with Cardiac MRI

**DOI:** 10.1371/journal.pone.0055424

**Published:** 2013-02-01

**Authors:** Bastiaan J. van Nierop, Hans C. van Assen, Elza D. van Deel, Leonie B. P. Niesen, Dirk J. Duncker, Gustav J. Strijkers, Klaas Nicolay

**Affiliations:** 1 Biomedical NMR, Department of Biomedical Engineering, Eindhoven University of Technology, Eindhoven, The Netherlands; 2 Signal Processing Systems, Department of Electrical Engineering, Eindhoven University of Technology, Eindhoven, The Netherlands; 3 Experimental Cardiology, Thoraxcenter, Erasmus MC, University Medical Center Rotterdam, Rotterdam, The Netherlands; Medical University Innsbruck, Austria

## Abstract

**Background:**

Left ventricular (LV) and right ventricular (RV) function have an important impact on symptom occurrence, disease progression and exercise tolerance in pressure overload-induced heart failure, but particularly RV functional changes are not well described in the relevant aortic banding mouse model. Therefore, we quantified time-dependent alterations in the ventricular morphology and function in two models of hypertrophy and heart failure and we studied the relationship between RV and LV function during the transition from hypertrophy to heart failure.

**Methods:**

MRI was used to quantify RV and LV function and morphology in healthy (n = 4) and sham operated (n = 3) C57BL/6 mice, and animals with a mild (n = 5) and a severe aortic constriction (n = 10).

**Results:**

Mice subjected to a mild constriction showed increased LV mass (*P*<0.01) and depressed LV ejection fraction (EF) (*P*<0.05) as compared to controls, but had similar RVEF (*P*>0.05). Animals with a severe constriction progressively developed LV hypertrophy (*P*<0.001), depressed LVEF (*P*<0.001), followed by a declining RVEF (*P*<0.001) and the development of pulmonary remodeling, as compared to controls during a 10-week follow-up. Myocardial strain, as a measure for local cardiac function, decreased in mice with a severe constriction compared to controls (*P*<0.05).

**Conclusions:**

Relevant changes in mouse RV and LV function following an aortic constriction could be quantified using MRI. The well-controlled models described here open opportunities to assess the added value of new MRI techniques for the diagnosis of heart failure and to study the impact of new therapeutic strategies on disease progression and symptom occurrence.

## Introduction

Heart failure (HF) is a progressive syndrome in which the heart is no longer capable of pumping blood at a rate commensurate with the peripheral needs [1]. HF is an important cause of morbidity and mortality worldwide and results in a significant decrease in the quality of life [2]–[4]. In many patients HF results from sustained, systemic hypertension accompanied by a pressure overload of the left ventricle (LV) [5]. The heart initially adapts to this overload by means of hypertrophic growth. However, a broad range of concomitant maladaptive processes, including myocardial fibrosis, metabolic changes and a decreasing capillary density, eventually lead to HF [6]–[8].

Despite considerable progress, the mechanisms responsible for the transition from compensated hypertrophy to HF are still not completely understood [9]. In particular, the role of the right ventricle (RV) long received comparatively little attention as compared to the LV in research on various cardiac pathologies. Recently, however, there is growing awareness that RV function has an important impact on disease progression, symptom occurrence and exercise tolerance in HF patients [10]–[11].

The goal of this study was therefore twofold. First, to quantify longitudinal changes in LV and RV morphology as well as function during the transition from a healthy to a compensated or decompensated state of LV hypertrophy. Second, to investigate the interplay between LV and RV function during this process.

Here, the well-defined, reproducible mouse model of transverse aorta constriction (TAC) can play a prominent role [12]. Currently there is a lack of information on the long-term changes in RV function and its relation with deteriorating LV function in this model. Therefore, mice were studied with cardiac MRI both in a compensated stage of cardiac hypertrophy resulting from a mild TAC and during the transition towards a stage of HF after application of a severe TAC [13].

## Methods

### Ethics Statement

All animal experiments were performed according to the Directive 2010/63/EU of the European Parliament and approved by the Animal Care and Use Committee of Maastricht University (protocol: 2009-019).

### Animal model

In this study 11 weeks old male C57BL/6 mice weighing between 23 and 25 g were used. Animals were housed under standard laboratory conditions with a 12 h light/dark cycle and were maintained on a standard diet and had access to water *ad libitum*.

For MRI a total of 22 animals were randomly separated in a control group (n = 4), a group that was sham-operated (n = 3) and in two groups which underwent a surgically induced mild (n = 5) or severe (n = 10) transverse aortic constriction (TAC), resulting in LV pressure overload [12]–[13]. Briefly, mice were anesthetized with 2.5 vol% isoflurane in 0.2 L/min O_2_ and 0.2 L/min medical air and intubated for mechanical ventilation. Animals were placed on a heating pad to maintain body temperature at 37°C. Buprenorphine (0.1 mg/kg s.c.) was administered for analgesia. Surgical procedures were performed using a stereo microscope (Leica M80). A small incision was made just lateral from the sternum above the first intercostal space. The aortic arch was exposed and tied off (6-0 silk suture) together with a 25G (Ø 0.50 mm) or 27G (Ø 0.42 mm) needle between the innominate artery and the left common carotid artery to induce a mild or severe TAC, respectively. The needle was immediately removed, restoring blood flow. The chest was then closed and the animals were allowed to recover in a 30°C recovery chamber. The sham-operation was identical, but without tightening of the ligation.

### MR examinations

Measurements were performed with a 9.4 T small animal MRI scanner (Bruker BioSpec, Ettlingen, Germany) equipped with a 740 mT/m gradient coil. A 72-mm-diameter quadrature transmit coil was used in combination with a 4 element phased-array receive coil (Bruker). Mice were anesthetized with isoflurane (4.0% for induction, 1.5–2.0% for maintenance) in medical air (0.4 L/min). The front paws were placed on ECG electrodes and a balloon pressure sensor was placed on the abdomen. Body temperature was maintained at 36–37°C with a heating pad and monitored with a rectal temperature sensor.

Cinematographic (cine) MR images were acquired using an ECG-triggered and respiratory-gated FLASH sequence, with the following parameters: pulse repetition time/echo time = 7/1.8 ms, number of signal averages = 6, α = 15, field of view = 3×3 cm^2^, matrix = 192×192, slice thickness = 1 mm, number of cardiac frames = 15–20. Measurements were performed in 2 long-axis and 5 short-axis planes, covering the LV from apex to base with interslice distance optimized for heart size.

Local cardiac function was measured from mid-ventricular short-axis tagged images. Tagging MRI was done using the FLASH sequence with a spatial modulation of magnetization (SPAMM) preparation module resulting in a sinusoidal modulation of magnetization that moves along with the cardiac tissue during the heart cycle. The preparation consisted of two Gaussian RF pulses (α = 45°, pulse width = 200 µs), separated by a gradient (duration = 200 µs) defining tag wavelength (0.5 mm) and orientation. Total duration of the preparation module was 2.7 ms. Tagged images were recorded with a reduced matrix of 192×96 (frequency×phase encoding) and reconstructed on a 384×384 matrix for data analysis. Tags were applied in horizontal and vertical directions and with 180° phase shift for complementary SPAMM (CSPAMM) reconstruction [14]. Total examination time was approximately 2 hours.

### Study protocol

**Figure 1:**
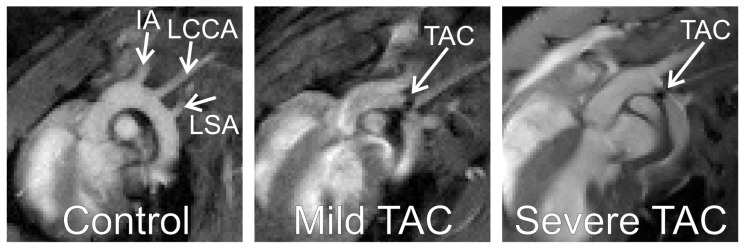
End-diastolic MR images of the aortic arch. Examples of MRI scans through the aortic arch in (left) a control, (middle) mild TAC and (right) severe TAC mouse. Indicated are (IA) the innominate artery, (LCCA) left common carotid artery, (LSA) left subclavian artery, and the transverse aortic constriction (TAC).

MRI measurements were performed at 1, 2, 4, 7, 10 and 13 weeks after surgery. Cine MR images were obtained at all time points. During the first MRI experiment cine MR images of the aortic arch were acquired to confirm correct positioning of the TAC (Fig. 1).

Tagged MR images were obtained at week 2 until week 10, at which time points all animals were in experiment, to determine the relationship between local strain changes and global cardiac morphology and function. Mice with a severe TAC were euthanized 10 weeks after surgery for animal welfare reasons. Immediately after the last measurements the anesthetized animals were killed by means of perfusion of the vascular bed with phosphate buffered saline (pH 7.4) infused via a needle penetrating the apex and exsanguination from the vena cava inferior. Next, the integrity of the aortic band was visually verified and lung wet weight (LuW) and tibia length (TL) were measured.

### Data analysis

The myocardial wall was segmented semi-automatically in the cine MR images using CAAS MRV FARM (Pie Medical Imaging, The Netherlands) to obtain LV and RV volumes, and the LV and RV ejection fractions (EF) [15]–[16]. Papillary muscles were excluded from the lumen. Wall thickening (WT) was defined as the percentage increase in wall thickness from end diastole to end systole. LV mass was calculated from end diastolic myocardial volume using a tissue density of 1.04 g/cm^3^ and was normalized to TL to obtain an independent measure of hypertrophy [17]. Pulmonary remodeling and edema were evaluated from the LuW/TL ratio and lung wet weight-to-dry weight ratio (ww/dw), respectively [18].

Local tissue motion was quantified from the tagged images using a method based on optical flow theory implemented in Mathematica 7.0 (Wolfram Research Inc., Champaign, IL) [19]. Briefly, the 180° phase shifted tagged images were subtracted from their complementary counterparts to obtain CSPAMM images. The phase of the tagging pattern was extracted by spectral filtering of the 1^st^ harmonic peak in k-space, using a Gabor filter bank [20]. Next, myocardial tissue displacements were computed from the extracted phases of the two time-series of orthogonal CSPAMM images, by solving a multi-scale version of the optical flow constraint equation. Finally, principal strains E1 and E2 were determined as read-out parameters for tissue deformation, as they report on radial wall thickening and circumferential wall shortening, respectively [21]–[22]. Strains were determined in end systole with end diastole as a reference. Strain analysis was performed in 4 segments according to AHA standards [23].

### Statistics

Data are expressed as mean ± standard deviation (SD). Changes in LV and RV volumes and EF, LV mass/TL, heart rate, respiratory rate, bodyweight (BW), WT and strains were tested for statistical significance with an ANOVA for repeated measures with time and group as factor, followed by the Bonferroni post-hoc test when appropriate. In case of interaction between time and group, the effect of time was tested separately per group. Changes in LuW/TL and heart weight/TL were tested for statistical significance with a 1-way ANOVA, followed by the Bonferroni post-hoc test. For survival analysis additional data from healthy (n = 48), mild TAC (n = 2) and severe TAC mice (n = 89) available from our laboratory was included. Differences between Kaplan-Meier survival curves were tested for statistical significance by means of Log Rank analysis. Calculations were performed using SPSS 19.0 (SPSS Inc., Chicago). For all tests the level of significance was set at α = 0.05.

## Results

### Experimental groups

**Figure 2:**
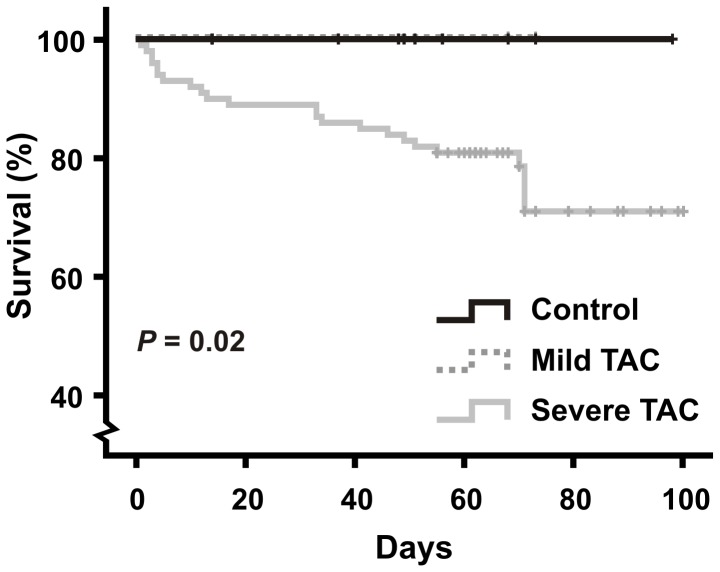
Kaplan-Meier survival curves. Kaplan-Meier analysis was performed based on survival data of a large cohort of healthy mice (n = 55), mild TAC (n = 5) and severe TAC mice (n = 99) available from our laboratory. Log Rank analysis showed a significant difference in survival between the severe TAC mice as compared to the control and mild TAC mice (*P* = 0.02).

Survival analysis performed on a large cohort of mice (Fig. 2) showed a significant difference (*P* = 0.02) in survival rate of the severe TAC mice, as compared to the control and mild TAC mice. In particular, all animals that underwent MRI in the control, sham-operated and the mild TAC group completed the experimental protocol. Possible differences in cardiac function or morphology between control and sham-operated mice were assessed in terms of LVEF and LV mass/TL (data not shown). No significant differences were detected (*P* = 0.25 and *P* = 0.76, respectively). Therefore, data of control and sham-operated groups were pooled for further analyses. One mouse with a severe TAC died within 60 min after surgery. The other animals recovered well from surgery. In this group, three mice died in the period 4–10 weeks after surgery, presumably due to acute decompensated HF or arrhythmias.

Table 1 lists general physiological parameters for the experimental groups. No significant changes over time or differences between groups were detected for heart rate and respiratory rate (*P*>0.05 in all cases). BW increased significantly during the course of the experiment (*P*<0.001), but no differences between groups were detected (*P*>0.05). BW in the severe TAC group displayed a decreasing trend at 10 weeks, which however did not reach statistical significance as compared to 7 weeks after surgery (*P*>0.05).

**Table 1:** General animal characteristics.

	Weeks	1	2	4	7	10	13
**HR**	Control	528±29	532±30	524±33	532±28	534±34	536±21
	Mild TAC	-	520±30	544±30	547±35	560±48	556±41
	Severe TAC	600±31	592±51	529±61	530±31	561±33	-
**Resp**	Control	74±6	86±23	89±8	90±10	90±12	89±9
	Mild TAC	-	82±17	77±9	81±17	74±16	95±14
	Severe TAC	110±15	88±9	81±18	89±12	87±12	-
**BW**	Control	24.4±0.8	25.4±1.3	26.0±1.2	27.0±1.3	27.8±1.6	28.9±1.8
	Mild TAC	-	26.9±2.2	27.6±2.4	28.0±2.0	29.6±1.7	28.4±0.9
	Severe TAC	24.5±0.9	25.2±1.1	26.5±1.2	27.3±1.2	25.8±0.7	-

General characteristics of the control animals and mice with a mild and severe constriction. Indicated are the time points relative to the time of surgery [weeks], the heart rate (HR) [min^−1^] and respiratory rate (Resp) [min^−1^] during the MR examination, and the body weight (BW) [g].

### Impaired LV function and hypertrophy

**Figure 3:**
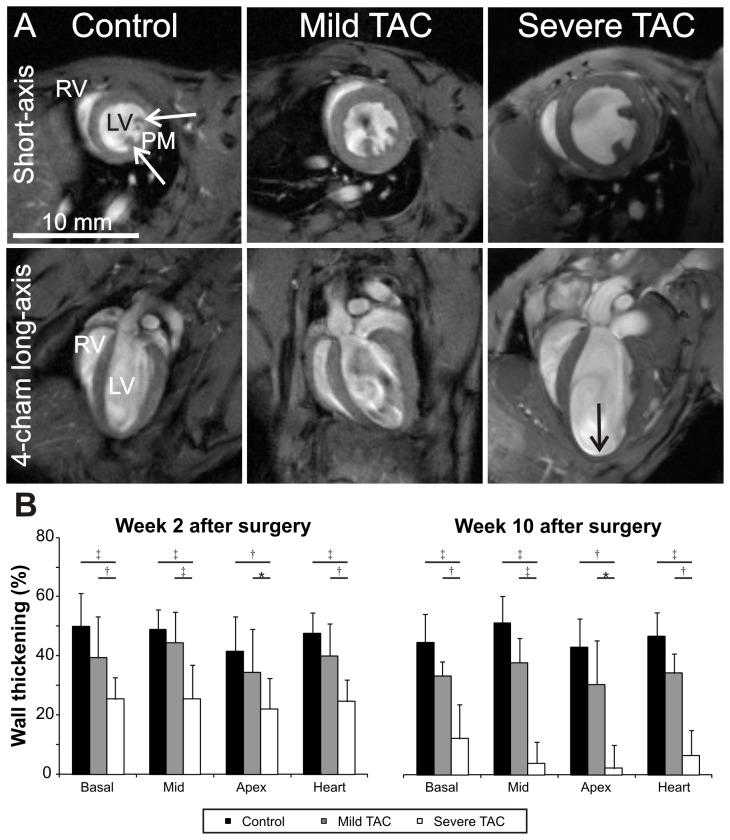
End-diastolic MR images. Representative end diastolic short-axis and long-axis images from control mice and mice subjected to a mild and severe aortic constriction 10 weeks after surgery (A). Indicated are the left ventricle (LV), right ventricle (RV), the papillary muscles (PM) and decreased apical wall thickness in the mouse with a severe TAC (↓). Corresponding movies can be found in the supplementary material. Wall thickening (WT) in the experimental groups at 2 and 10 weeks after surgery (B). At 2 weeks, WT had decreased in all sections of the heart in mice with a severe TAC as compared to controls (*P*<0.01 in all cases), but the decrease in mild TAC mice did not reach statistical significance (*P*>0.05). At 2 weeks, WT in the animals with a mild constriction was significantly different from the mice with a severe constriction in all portions of the heart (*P*<0.05, in all cases). No changes over time were detected in the control and mild TAC mice (*P*>0.05, in all cases), whereas WT decreased in all portions of the severe TAC hearts (*P*<0.05, in all cases).

Figure 3A shows representative short-axis and long-axis cine MR images obtained in the different experimental groups 10 weeks after surgery. Increased wall thickness was observed in all mice subjected to TAC, while apical aneurysms were only noted in severe TAC.

**Figure 4:**
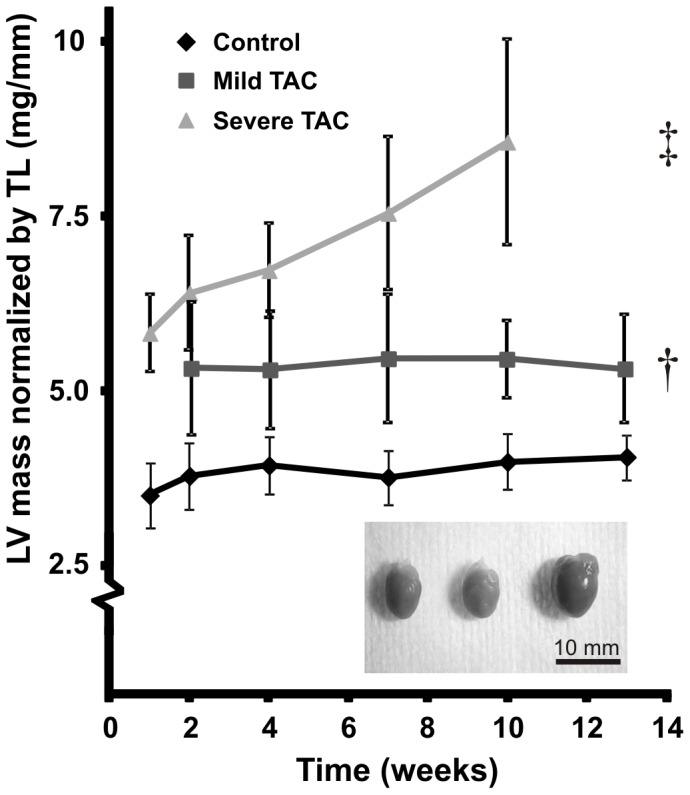
Left ventricular mass. LV mass normalized to tibia length (TL) in control, mild and severe TAC mice as a function of time. Cardiac mass slightly increased in response to a mild constriction as compared to controls (*, *P*<0.05) and progressively increased in response to a severe constriction (‡, *P*<0.001). Mean and SD per time point are denoted by the corresponding symbol and error bars. The inset shows a photograph of (left) a control, (middle) mild TAC and (right) severe TAC heart 10 weeks (severe TAC) and 13 weeks (control and mild TAC) after surgery.

**Figure 5:**
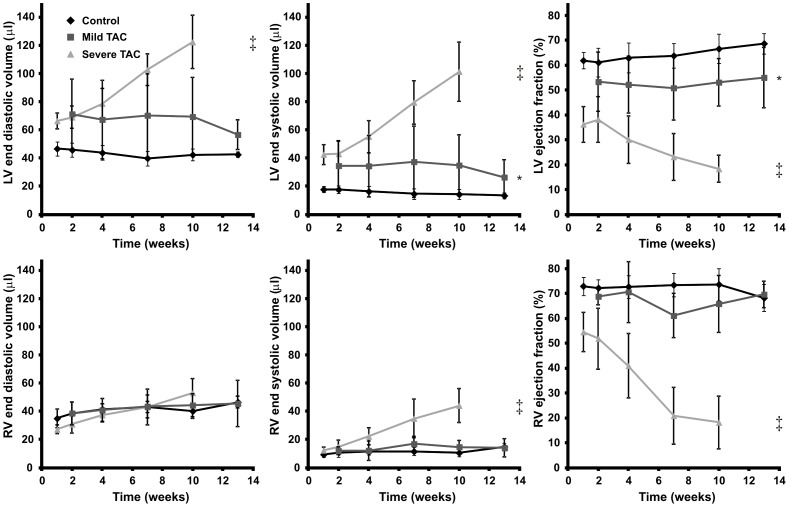
Left and right ventricular volumes. End diastolic volume in control, mild and severe TAC mice (left column), end systolic volume (middle column) and ejection fraction (right column) as a function of time for both the left ventricle (LV) (top row) and right ventricle (RV) (bottom row). End diastolic and end systolic volumes clearly show LV and RV dilation in the severe TAC mice, but not in the mild TAC mice as compared to the control animals. LV ejection fraction was slightly depressed in response to a mild constriction as compared to controls, and showed a progressive decline in time in the group with a severe constriction. RV ejection fraction remained unchanged in mice with a mild constriction of the aorta as compared to control mice, but showed a progressive decline in case of a severe aortic constriction. Mean and SD per time point are denoted by the corresponding symbol and error bars. Statistical differences as compared to the control group are indicated by * (*P*<0.05), † (*P*<0.01) and ‡ (*P*<0.001).

Mice with a mild TAC revealed a small increase of LV mass normalized to TL (5.4±0.7 mg/mm) as compared to controls (3.9±0.4 mg/mm, *P*<0.01) (Fig. 4). In these animals, a mild impairment of systolic function was apparent from a depressed LVEF (53±10%) as compared to controls (64±6%, *P*<0.05), a trend towards increased LV end diastolic volume (EDV) (69±23 µl) as compared to controls (44±5 µl, *P* = 0.06) and a slightly increased LV end systolic volume (ESV) (35±19 µl) as compared to controls (16±4 µl, *P* = 0.05) (Fig. 5). LV mass, EDV, ESV and EF did not change significantly over time in both groups (*P*>0.05).

In contrast, severe TAC resulted in a progressive increase of LV mass normalized to TL from 5.8±0.6 mg/mm in week 2 to 9.1±0.5 mg/mm in week 10 (*P*<0.001). As a result of the high pressure overload a significant reduction in EF (36±7%, *P*<0.001) was apparent by week 1. No further deterioration of EF occurred between 1 and 2 weeks after surgery (*P*>0.05). At later time points EF gradually decreased to 18±5% in week 10 (*P*<0.001). EDV and ESV remained virtually constant between week 1 and 2, but progressively increased afterwards (*P*<0.001). In addition, starting from 4 weeks after surgery akinesia of the apex was detected in a subset of the severe TAC group (n = 3) (black arrow, Fig. 3A) accompanied by apical aneurysms of the LV wall. Akinesia was quantified in terms of WT (Fig. 3B).

### Impaired RV function and lung remodeling

**Figure 6:**
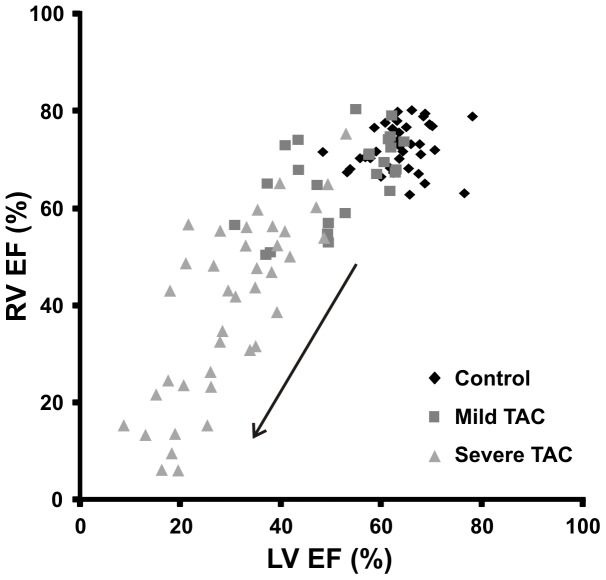
Left versus right ventricular ejection fraction. Relationship between left (LV) and right ventricular (RV) ejection fraction (EF) for all mice at all time points. The right ventricular ejection fraction progressively decreased (black arrow) in the severe TAC group only and that the changes in RVEF were preceded by a decline in LVEF apparent from the shift of the majority of the measurement points to the left.

Impaired LV function may induce lung remodeling and/or edema and subsequently RV failure [10]. Therefore, RV EDV, ESV and EF were also quantified over time (Fig. 5). RVEF progressively decreased from 56±10% to 18±11% (*P*<0.001) and RV ESV increased from 12±2 µl to 44±12 µl (*P*<0.001) in mice with a severe TAC. In contrast, RVEF in mild TAC animals (66±9%) was not depressed as compared to controls (72±5%) and RV EDV (42±8 µl) and ESV (14±5 µl) in mild TAC animals remained unchanged as compared to RV EDV (41±6 µl) and ESV (12±3 µl) in controls (*P*>0.05 in all cases). Figure 6 shows the relationship between RV and LVEF for all mice. RVEF was merely affected in the severe TAC mice and was preceded by a change in LVEF. This became apparent from a shift of the majority of the measurement points to the left in Figure 6, indicating that LVEF decreased first before deterioration of RVEF. Finally, pulmonary remodeling (Table 2) was observed in the severe TAC group when the mice were euthanized, indicated by an increased LuW/TL ratio as compared to controls and mild TAC (*P*<0.01), but no increase in lung water content was observed, indicated by the lung wet weight-to-dry weight ratio (*P*>0.05).

**Table 2:** Assessment of pulmonary remodeling and heart weight.

Group	LuW/TL [mg/mm]	ww/dw [-]	HW/TL [mg/mm]
Control	9.4±1.5	6.1±0.6	7.9±0.5
Mild TAC	10.4±2.0	6.8±1.8	10.3±2.1
Severe TAC	17.5±4.8^†^	5.6±0.7	13.6±2.3^‡^

An increased lung weight-to-tibia length (LuW/TL) ratio [mg/mm] indicated the presence of pulmonary remodeling in the mice with a severe constriction (†, *P*<0.01), but not in the mice with a mild constriction. No differences between groups were observed in the lung water content, indicated by a constant lung wet weight-to-dry weight ratio (ww/dw) (*P*>0.05). Post mortem whole heart weight-to-tibia length ratio was significantly increased in severe TAC mice (*P*<0.001), but the increase in the mild TAC mice did not reached statistical significance (*P*>0.05).

### Myocardial principal strains

**Figure 7:**
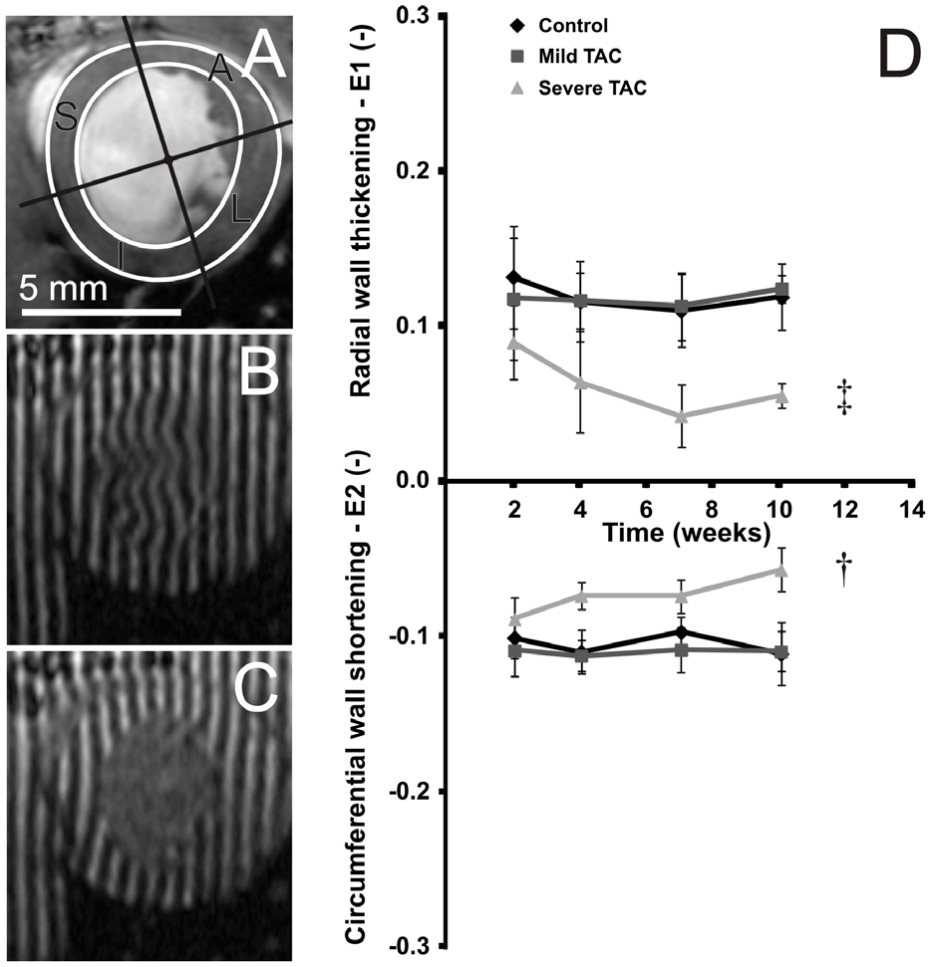
Left ventricular radial wall thickening and circumferential wall shortening. Figure 7D shows that radial wall thickening and circumferential wall shortening were identical between the control (0.12±0.03 and −0.10±0.02, respectively) and mild TAC mice (0.12±0.02 and −0.11±0.02, respectively) (*P*>0.05). Moreover, no effects of time were observed (*P*>0.05). In contrast, radial wall thickening was significantly decreased in the mice with a severe TAC as compared to control mice (*P*<0.001) and showed a decrease from 0.09±0.02 in week 2 to 0.06±0.01 in week 10 (*P*<0.05). Circumferential wall shortening was also significantly decreased in mice with a severe TAC as compared to control mice (*P*<0.01) and showed a decrease from −0.09±0.01 in week 2 to −0.06±0.01 in week 10 (*P*<0.05).

Figure 7 shows an example of a short-axis cine image (A) and corresponding tagged images in end diastole (B) and end systole (C) in a mouse with a severe TAC 10 weeks after surgery. Myocardial principal strains E1 and E2 were determined as read-out parameters for radial wall thickening and circumferential wall shortening, respectively. Analysis of the principal strains in the four segments (see Fig. 7A) revealed no marked, regional differences between groups (data not shown), and were therefore reported for the myocardium as a whole.

## Discussion

In this study we investigated the evolution of RV and LV function in well-controlled mouse models of compensated hypertrophy and decompensated HF as induced by two different degrees of transverse aortic constriction (TAC), using cinematographic and tagging MRI [13].

There are a number of important findings to this study. First, mice with a mild TAC revealed myocardial hypertrophy and only a slightly depressed LVEF consistent with a state of compensated hypertrophy. Second, mice subjected to a severe TAC showed progressive LV hypertrophy, increased LV volumes and a drastic decline in LV function in accordance with a condition of HF. Third, myocardial principal strains were significantly reduced in severe TAC mice as compared to controls and progressively decreased over time. Fourth, changes in RV volumes and EF could be quantified in TAC mice using cardiac MRI. Fifth, the progressive deterioration of LV function in severe TAC mice was followed in time by worsening of RV function and severe pulmonary remodeling, two important hallmarks of congestive left ventricular pump failure [10], [18], [24].

LV mass, end diastolic and end systolic volumes were slightly elevated at 2 weeks after mild TAC, after which these variables remained essentially constant. RV volumes and EF in mild TAC mice, however, were unchanged as compared to control mice. The severe TAC mice showed a progressive increase in both end diastolic and end systolic volumes accompanied by a decline in LVEF. In these mice also a marked increase was found in RV end systolic volume as compared to the control mice resulting in a deteriorating RVEF. The absence of RV dilation, RV end diastolic volumes remained unchanged, may point to impaired contractile properties rather than dilation as a cause for the impaired RV function. These mice likely also developed profound pulmonary remodeling, as indicated by an increased LuW/TL ratio (17.5±4.8 mg/mm) as compared to controls (9.4±1.5 mg/mm) (*P*<0.01). However, no differences in lung water content were observed, indicated by a constant lung wet weight-to-dry weight ratio (*P*>0.05). This is in line with recent evidence suggesting that increased lung mass secondary to LV failure in TAC mice is caused by pulmonary remodeling with an increased percentage of fully muscularized vessels, vascular and lung fibrosis, myofibroblast proliferation, and leukyocyte infiltration, but not by an increased lung water content [18]. Finally, the severe TAC mice showed a progressive increase in LV mass. Moreover, in a subset of these mice extreme apical wall thinning and akinesia was observed. The underlying mechanisms responsible for the formation of these apical aneurysms in response to pressure overload-induced hypertrophy, however, remain to be determined. Taken together, the mild TAC mice revealed a condition of compensated hypertrophy, whereas the severe TAC mice developed overt congestive biventricular failure.

Awareness is growing that RV function has an important impact on symptom occurrence, disease progression as well as exercise tolerance in various cardiac pathologies [10]. Pulmonary hypertension is one of the most prominent causes of RV failure and is often due to LV pathologies [10]. Recent evidence suggests that the RV and LV are categorically different [10], [25]. For example, both ventricles originate from different progenitor cells and sites during cardiac morphogenesis, have a different morphology and show important differences with respect to the expression of regulatory proteins in response to stressors as ischemia and hypertension. Thus, RV failure cannot be understood by straightforward extrapolation of the knowledge about LV failure. Although surgically induced TAC in mice has been extensively used as a model to study pressure overload induced LV hypertrophy and failure, data on RV function in this model is scarce [12]–[13], [26]–[29]. Since RV failure in severe TAC results from LV pathology, it could also be used as a highly relevant model to study RV adaptations to LV hypertrophy and failure. Quantification of murine RV function using echocardiography is not straightforward due to the complex shape and motion of the RV. The alternative use of conductance catheters is invasive and cannot yield information on cardiac mass [30]–[31]. In contrast, cardiac MRI offers the possibility to quantify both RV function and potentially also RV mass and could therefore well be used to study the interplay between both ventricles, as was done in this study [16].

Cardiac strains were quantified in terms of the 2D principal strains from tagged MR images using a method based on optical flow theory. While myocardial principal strain E1 is mainly oriented in the radial direction, E2 coincides with the circumferential direction [22]. No differences were observed in both principal strains between the control and mild TAC group. Moreover, no time effect was observed in both groups, in line with the essentially constant EF. Furthermore, no differences in WT were observed between healthy and mild TAC mice, in agreement with the absence of differences in radial wall thickening E1. On the other hand, the severe TAC mice showed a significant decline of the radial wall thickening and circumferential wall shortening from week 2 to 10, which was paralleled by a large drop in EF. Finally, the lowering of radial wall thickening was reflected in a reduction in WT.

The effects of both passive and active cardiac tissue mechanics on the transition from hypertrophy to heart failure gain increased interest [32]–[33]. Therefore, we investigated whether early strain changes precede late changes in global cardiac morphology or function during the development of HF. However, such an effect was not observed. Instead, changes in WT and myocardial strains were in accordance with alterations in global cardiac parameters. These findings suggest that the added value of local strain analysis is more evident when heterogeneous myocardial contraction is anticipated, for example in the infarcted heart, as compared to pathologies with an essentially homogeneous contraction pattern, as studied here.

The septum is believed to contribute to both LV and RV function in the normal and diseased heart, although the precise mechanisms are not fully understood [30]. We therefore determined the relationship between septal principal strains and RVEF for all groups. However, in our data we did not find any clear relationship between these parameters (r<0.30 in all cases). Although approximately 24% of RV ejection depends on LV contraction, the majority of RV ejection results from longitudinal shortening of the RV during systole [10]. Most likely 3D strain analysis in the septal wall will be required to determine the contribution of septal motion on RV ejection during deterioration of LV function.

The disease progression reported in this study compares well with previous data, despite the fact that the phenotype resulting from TAC surgery may vary depending on mouse strain and the surgical technique used [29]. Rothermel *et al.* induced a mild (Ø 0.42 mm) and a severe TAC (Ø 0.38 mm) in 6–8 weeks old, male C57BL/6 mice [34]. Animals with a mild TAC developed a significant increase of LV mass as compared to healthy mice, but were clinically indistinguishable. By contrast, mice subjected to a severe TAC developed signs of end-stage HF within 3 weeks. In our study 11 weeks old mice were used, necessitating the use of larger needle diameters, both for the induction of the mild and the severe TAC.

Cardiac MRI is an important clinical tool for HF diagnosis [35]. Currently, imaging biomarkers for the characterization of HF are often restricted to cardiac pathological anatomy (LV mass) and function (LVEF). However, the management of patients with hypertrophy and HF may significantly benefit from additional imaging read-outs reporting on the presence of fibrosis, a decreasing capillary density, or changes in cardiac metabolism. A range of MR techniques have recently become available for this purpose, including cardiac T_1_ mapping [36]–[37], quantitative perfusion MRI [38]– and MR spectroscopy for the heart [40]–[41]. The well-controlled disease model presented in this paper might prove an important preclinical step to assess the added value of these novel imaging read-outs for HF diagnosis. Since the RV is gaining increasing attention as a potential therapeutic target, it might also be of great interest to study the effects of new therapeutic strategies on both LV and RV function in TAC mice using MRI [25]. One example is the inhibition of small molecule histone deacetylase, which has been shown to block myocardial remodeling in various HF models [42]. Such studies could also take into account potential improvements in exercise capacity related to RV function.

There are some limitations to this study. A valuable comparison of LV and RV volumes and function with, for example, conductance catheters measurements was not made. However, it was anticipated that this would require a large cohort of mice, since catheter measurements in mice are terminal. Instead, the number of mice required was minimized by choosing a longitudinal study design with readouts from non-invasive imaging. The experimental variation in for example LV mass, LVEDV and LVESV increased during the course of the experiment, in particular in the severe TAC group, but was comparatively small at the start of the experiment. This suggests that within group differences in systolic pressure gradient immediately after TAC were small and that the observed variation resulted from inter animal differences, but we cannot fully exclude some variation due to small differences in pressure gradient. Although the number of mice in the sham and mild TAC groups was limited, the longitudinal study design generally is more efficient and results in increased statistical power as changes over time are assessed within the same animals.

## Conclusions

In this study longitudinal MRI measurements were performed in mice subjected to a mild or severe TAC. The mice with a mild TAC developed compensated hypertrophy, whereas the mice with a severe TAC developed congestive HF. A decline in RV function was observed following the progressive deterioration of LV function, relevant for many cases where RV failure develops secondary to LV pathologies. The well-controlled aortic banding model of HF described here therefore opens opportunities to assess the added value of various new MR imaging techniques for the diagnosis of HF, to study the impact of new therapeutic strategies on disease progression and symptom occurrence in the RV and LV, and to assess the effects of pharmacological or mechanical LV unloading on the RV. Such studies might eventually lead to improvements in care for patients suffering from pressure overload-induced HF.

## Supporting Information

Movies S1 **Cinematographic MRI: Control mice.** Representative end diastolic short-axis and long-axis images from a control mouse.
(PPT)

Movies S2 **Cinematographic MRI: mild TAC mice.** Representative end diastolic short-axis and long-axis images from a mild TAC mouse.
(PPT)

Movies S3 **Cinematographic MRI: severe TAC mice.** Representative end diastolic short-axis and long-axis images from a severe TAC mouse.
(PPT)
