# Supplementary material for: Phenotyping of Left and Right Ventricular Function in Mouse Models of Compensated Hypertrophy and Heart Failure with Cardiac MRI
Source: PLoS One. 2013 Feb 1;8(2):e55424. doi: 10.1371/journal.pone.0055424 (PMC3562232; doi:10.1371/journal.pone.0055424)
Supplement: Movies S3 — Cinematographic MRI: severe TAC mice. Representative end diastolic short-axis and long-axis images from a severe TAC mouse. (PPT) [file pone.0055424.s003.ppt]

## Slide 1
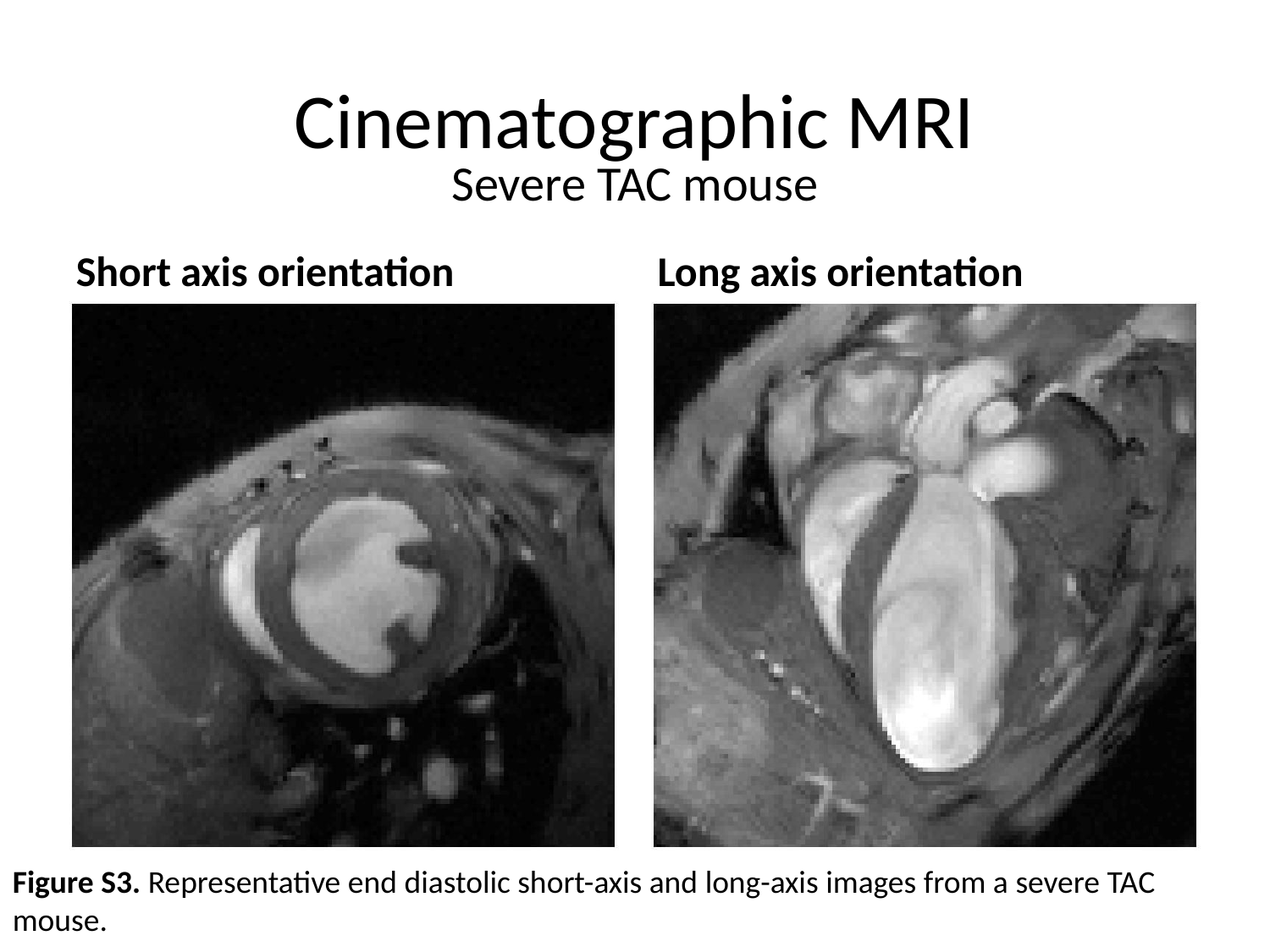

# Cinematographic MRI
Severe TAC mouse
Short axis orientation
Long axis orientation
Figure S3. Representative end diastolic short-axis and long-axis images from a severe TAC mouse.
